# Alterations in cytoskeletal and immune function-related proteome profiles in whole rat lung following intratracheal instillation of heparin

**DOI:** 10.1186/1465-9921-8-36

**Published:** 2007-05-08

**Authors:** Amir A Gabr, Mathew Reed, Donna R Newman, Jan Pohl, Jody Khosla, Philip L Sannes

**Affiliations:** 1 Department of Molecular Biomedical Sciences, Center for Comparative Molecular Medicine and Translational Research, College of Veterinary Medicine, North Carolina State University, Raleigh, NC, USA; 2 Microchemical and Proteomics Facility, Winship Cancer Institute, School of Medicine, Emory University, Atlanta, GA, USA

## Abstract

**Background:**

Heparin has been shown to modify fundamental biologic processes ranging from blood coagulation and cell proliferation to fibrogenesis and asthma. The goal of this study was to identify specific or broad biologic responses of the rat lung to intratracheal instillation of heparin by targeted proteomic analysis.

**Methods:**

Rats were given either aerosolized 500 μg heparin in 250 μl saline or saline alone. Lungs were harvested at 0, 24, or 96 hours post-treatment and isolated proteins analyzed by two-dimensional gel electrophoresis. Proteins which increased and decreased significantly in treated groups above controls were then selected for identification by mass spectrometry.

**Results:**

Although heparin treatments resulted in a general reduction in cytosolic protein expression, there were significant increases within members of discrete groups of proteins. At 24 hours, proteins which function in cytoskeletal organization and in calcium signaling were up-regulated between 2- and 27-fold above baseline and untreated controls. Increased proteins include annexins V and VI, septin 2, capping G protein, actin-related protein 3, moesin, RhoGDP dissociation inhibitor, and calcyclin. A group of proteins relating to immune response and tumor suppressor function were either up-regulated (tumor suppressor p30/hyaluronic acid binding protein-1, Parkinson disease protein 7, proteosome 28 subunit/interferon-γ inducible protein, and proteosome subunit macropain α-1) or strongly down-regulated (transgelin). At 96 hours, most proteins that had increased at 24 hours remained elevated but to a much lesser degree.

**Conclusion:**

These cumulative observations demonstrate that whole lung heparin treatment results in significant up-regulation of selected groups of proteins, primarily those related to cytoskeletal reorganization and immune function, which may prove to be relevant biomarkers useful in analysis of lung exposures/treatments as well as in system biology studies.

## Background

Proteomic studies performed on whole organs can provide a useful, broader perspective from which to view a biological event [1,2]. As applied to the lung, this approach may provide important information on functional groups of proteins, a prerequisite for systems biology studies, while supporting dynamic modeling. Ultimately, pulmonary function or its disturbance can be thought of as the result of the interactions of a dynamic group of proteins originating from a diverse population of cells varying in, activation states and levels of maturity, which collectively constitute the lung proteome [3]. Heparin, a model heparan sulfate proteoglycan (HSPG), has been previously shown to reduce or inhibit proliferation and gene and protein expression in isolated rat lung type II alveolar cells [4-6]. Similarly, heparin and/or heparan sulfate have been shown to reduce proliferation in lung fibroblasts [7-9] and lung smooth muscle cells [10,11]. The current proteomic study was undertaken to expand investigation into the effects of heparin-like molecules on whole lung, in vivo. Normal lung contains large amounts of HSPGs, many of which are heavily sulfated. Syndecan, an HSPG spanning the cytoplasmic membrane, has a sulfated ectodomain comprised of a portion of the core protein and a variable number of glycosaminoglycan (GAG) side chains that can be enzymatically shed, both constitutively at low levels and at much higher levels during inflammation and tissue remodeling [12]. Heparin's anti-coagulant activity is well established, but more recently it has been appreciated for its antiproliferative effects, as noted above, which can prevent lung damage due to hypoxia [11], bleomycin-induced fibrosis [13] and cancer [14]. These effects have been shown to be a function of the sulfated nature of these molecules, as desulfated or otherwise modified forms are not as effective [4-6,9,10]. Heparin has also been shown clinically to inhibit specific asthmatic responses in humans and prevent bronchoconstriction in exercise-induced asthma by mechanisms not fully understood [15-17]. It has been used experimentally as a model component of extracellular matrix and cell surface shedding [4-6,16].

The goal of this study was to identify specific proteomic responses of normal rat lungs exposed to aerosolized heparin using 2-dimensional gel electrophoretic separation and mass spectrometry (MS). Proteins so defined could then serve as potential biomarkers or indicators of responses to changes in the lung macro- and/or microenvironments that might be expected to increase during cell surface ectodomain shedding as in injury or disease, in the context of a lung systems biology approach. Although it is not intended to serve as a complete catalogue of the whole rat lung proteome, the results could be a useful reference to future proteomic studies on rat lung.

## Methods

### In-vivo heparin treatments

Two groups of specific-pathogen-free (SPF) rats were used in this study. The first group, analyzed as preliminary data for the subsequent study, consisted of Fischer rats (n = 9) (Charles River, Wilmington, MA) in 3 groups of 3 animals each: untreated controls, heparin-treated and sacrificed after 24 hours, and heparin-treated and sacrificed after 96 hours.

The second group consisted of Fischer rats (n = 15)(Harlan, Indianapolis, IN) in 5 subgroups of 3 animals each: untreated, saline/vehicle control 24 hours, heparin-treated 24 hours, saline/vehicle control 96 hours, and heparin-treated 96 hours. One untreated control (baseline/reference) was sacrificed at 0, 24, and 96 hours. Vehicle controls and heparin-treated animals were sacrificed at 24 and 96 hours.

Rats weighing between 250 and 285 grams were given intraperitoneally a mixture of 30 mg Ketaset (Fort Dodge, Fort Dodge, IA) and 6 mg X-ject (Burns Veterinary Supply, Inc., Westbury, NY) to induce anesthesia 15 min prior to intratracheal administration of a single aerosolized dose (250 μl) of normal saline or of a 2 mg/ml solution of high molecular weight heparin, 13,500–15,000 MW, (Calbiochem, San Diego, CA) using a MicroSprayer intratracheal aerosolizer (Penn-Century, Philadelphia, PA) to optimize the distribution of heparin throughout the lung. According to the manufacturer, this generates 25–30 μm sized particles, which should be capable of reaching first order (primary) bronchioles. At 24 hours or 96 hours post-treatment, the experimental and control animals were euthanized with an overdose of sodium pentobarbital. The lungs were perfused with saline until white to remove blood and minimize albumin interference in later procedures, then surgically removed from the pulmonary cavity. Pulmonary airways (visible bronchi) were separated by blunt dissection from the parenchyma (small airways and alveolar regions) and the latter flash frozen in liquid nitrogen and stored at -80°C for later analysis. In some cases, the apical lobe of the right lung was tied off and insufflated with 2% paraformaldehyde in 0.1 M phosphate buffered saline for 1 hour. Sections were dehydrated with graded alcohols and infiltrated with xylenes followed by paraffin. Paraffin embedded tissue blocks were cut into 7 μm sections on a standard microtome and prepared for routine histology or immunostaining. Rabbit anti-annexin V (FL-319; sc-8300, Santa Cruz Biotech, Santa Cruz, CA) was applied at 1:100 dilutions overnight and the antigen-antibody complex visualized with a VectaStain Elite Peroxidase kit (Vector Laboratories, Burlingame, CA).

### Protein sample preparation

Frozen lung parenchyma was pulverized using a specialized mortar and pestle (Fisher Scientific, Suwanee, GA) under liquid nitrogen. Equal amounts (250 mg) of powdered lung were suspended in a urea-containing buffer containing 20 μg/ml RNase-A, 100 μg/ml DNase-1 inhibitor (Invitrogen, Carlsbad, CA), 1% ampholytes (pH 3–10), and 50 μM dithiothreitol (BioRad, LaJolla, CA). Protein concentrations were measured by chemiluminescence using Protein EZ-Quant (Invitrogen), with 100 μg total lung lysate loaded per gel. All samples were diluted in 6.25 M urea and 2.3 M thiourea buffer to a concentration of 1 mg/ml and stored frozen at -80°C. First-dimensional Readystrips, pH 5–8, 11 cm (BioRad) were used for all samples based on preliminary data that suggested the majority of dynamically regulated proteins focused between pH 5 and pH 8. Samples were applied to strips and equilibrated for 12 hours at 50 V on a Protean X IEF focuser (BioRad). Samples were isoelectrically focused at 250 V for 15 minutes. Voltage was increased by two 500 V ramp increases until achieving 1500 V, held at 1500 V for 2 hours, and then increased in rapid steps to 8000 V until complete isoelectric focus was achieved at 55 kVH.

### Second-dimension electrophoresis and staining

Bis-Acrylamide gels (12.5%) with stackers (4%) were hand-cast in a 16 cm × 18 cm gel slab Multicasting System (Hoefer Scientific, Cambridge, MA). Gels were allowed to polymerize overnight with n-butanol overlays. First-dimension electrofocused strips were alkylated in 0.25% iodoacetamide (BioRad) for 20 minutes on an orbital shaker, rinsed with 1× Tris-Glycine-SDS running buffer, and then carefully laid onto second-dimension gels. A layer of 0.5% agarose containing bromophenol blue was poured to keep strips in place. Multiple gels were run simultaneously (to minimize group run variation) at 200 V for 6 hours, rinsed for 1 minute in 250 ml of ultra-pure water twice, and fixed overnight in 10% methanol, 5% glacial acetic acid on a horizontal shaker. Gels were incubated in Sypro-Ruby stain (Molecular Probes, Carlsbad, CA) in the dark for 16 hours.

### Image capture and analysis

Gel images were captured with an Epi Chemi UVP scanner supported by a 16 bit CCD camera for enhanced high resolution images (BioImaging Systems, Upland, CA). Aperture width and exposure time were adjusted so that only the most abundant spots on the gels reached saturation (pixel intensity was determined by the software). In addition, gels were normalized based on total protein fluorescence per gel as well as reference spot fluorescence comparison. The same settings were used for all gels. Acquired gel images were analyzed using PDQuest software (BioRad), transformed to adsorption images (dark spots against light background), cropped, and size-adjusted across all images. The effects of occasional streaking were minimized by during the analysis with the application of Guassian equations to the raw images with the PDQuest software. Spot detection parameters were optimized on a single gel representative of each individual sample, then normalized among the sub-group, and finally normalized across groups. Areas of interest were assigned to the program for further magnification. Images in each set were matched and a representative map was generated for each individual and subsequently for each sub-group per time point. Correlation coefficients of variance were calculated for each spot within sub-groups and compared with the mean correlation coefficient of variance in the software program, which then assigned significance values to each spot. Only spots with optical densities that were significantly changed by heparin (based on the Mann-Whitney statistical technique) were further analyzed for the purposes of this study.

### Spot selection, digestion, and protein analysis

After selection of the most dynamically-regulated spots, gel plugs were manually excised from replicate gels (n = 3), placed in a 96-well plate, trypsin-digested overnight on an automated digester, and placed on zip-plates (Millipore Bioscience, Bedford, MA) to remove any salts that could adversely affect results. Bovine albumin was added to the Maldi plate as a positive control for MS detection. Replicate samples and control proteins were resuspended in 5 μl of 30% acetonitrile/0.1% trifluoroacetic acid. One μl of each sample was spotted twice, allowing the spot to air dry completely each time. Matrix (alpha-cyano-4-hydroxycinnamic acid, Agilent Technologies, Palo Alto, CA) (0.5 μl) was overlaid on each spot. An ABI-4700 Proteomics Analyzer MALDI-time of flight/time of flight (TOF/TOF) mass spectrometer (Applied Biosystems, Foster City, CA) was used for the analysis. The instrument was set to acquire an initial MS spectrum followed by up to 15 MS/MS spectra for each spot. The instrument was internally calibrated using a mixture of 6 peptides. A default "plate" calibration was performed based upon 6 calibration spots distributed on the plate, resulting in accuracies better than 100 ppm. For the analysis of sample spots, the instrument attempted to perform an internal calibration for the individual spots using the tryptic autodigest fragments, which resulted in accuracies better than 20 ppm. If the instrument was unable to perform the internal calibration, the default "plate" calibration was applied to the sample spot. The MS and MS/MS data were processed by GPS Explorer, version 2.0 (Applied Biosystems), and submitted to our in-house MASCOT search engine (Matrix Science). The NCBI non-redundant database was searched with tolerances set to 150 ppm for the MS spectra and 0.5 kDa for the MS/MS spectra. GPS Explorer then processed the search results from MASCOT.

## Results

### Lung histology

**Figure 1:**
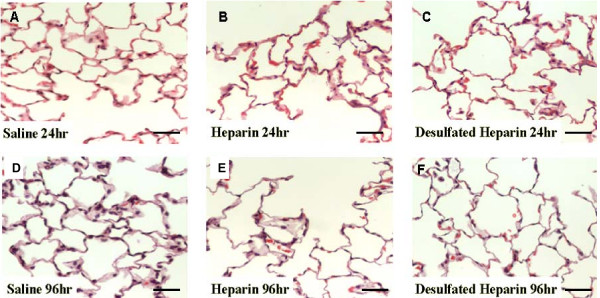
Light micrographs depicting representative, random histologic fields from the various treatment groups indicated (A-D). No significant changes in lung architecture, cell infiltrates, or cell numbers were apparent with any of the treatments and the respective time points. Magnification ×250. Bar = 40 μM.

Tissues prepared for morphologic examination indicated no gross or microscopic alterations in lung architecture due to the instillation of heparin at any of the time points examined (Figs. 1A–D).

### Preliminary experiment analysis

Initially, 4128 spots were detected within the preliminary data set using PDQuest software and liberal parameters. Parameters of very high stringency in combination with a most conservative threshold then allowed the software to reassign 1682 spots for comparison. The software identified 551 differentially-expressed (increased/decreased) proteins, not all of which were obvious to the unaided eye. Of those, 54 protein spots were selected as the highest priority candidates based on the magnitude of change in expression and were further analyzed by mass spectrometry.

Particularly large or diffuse spots, such as those likely containing multiple proteins or representing albumin and/or transferrin, were not considered good candidates for quantitative analysis by MS and were excluded from further analysis.

### Controlled experiment analysis

**Figure 2:**
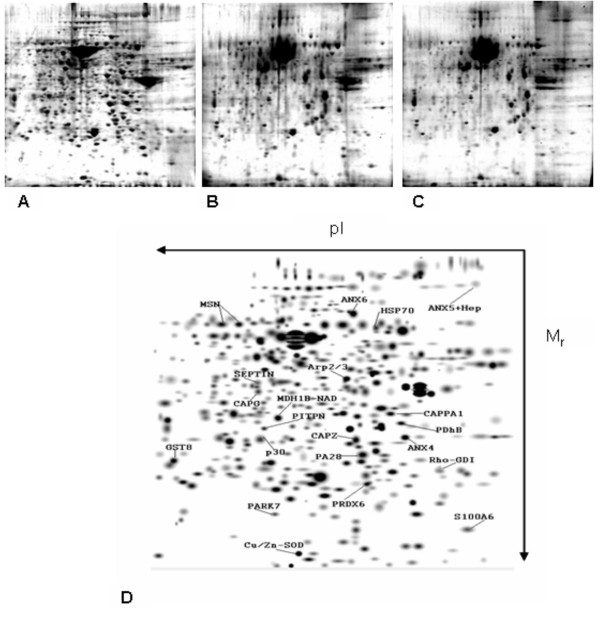
Protein maps representative of each of the treatment sub-groups analyzed in side by side comparison and filtered baseline reference map annotation. **A**. Control treatment, **B**. heparin 24 hours, **C**. heparin 96 hours, and **D**. a computer analyzed, 2D Gaussian filtered lung reference map with the identified protein spots, separated based on pI and molecular weight (Mr). Note repeatability between sub-groups.

Across the controlled experiment data set, 3103 spots were detected using the software at liberal parameters. With more stringent parameters, the software reassigned 942 spots for further comparison. Technical stringency indicated almost identical protein migration patterns (Figs. 2A–D) between experiments (especially Heparin treatments), hence achieving a higher degree of analytical confidence. Next, the software was able to identify 377 differentially-expressed proteins within the spots, which were again examined in a hierarchical manner.

### Analysis of combined experiments

There were no visual or analytical differences between untreated, saline 24 hours, and saline 96 hours sub-group samples (CV% range of 0.01–0.1). Comparisons by the imaging software of images from replicate samples within experimental subgroups (CV% of 0.05) and between maps of untreated controls and vehicle control groups indicated no statistically-significant differences (within 95% confidence levels). The repeatability of patterns between sub-groups enabled reliable statistical analysis of changes in protein expression/spot detection, as demonstrated in Figure 2A–D. Images with equivalent statistical strengths across replicates within subgroups were aligned to generate reference templates per subgroup which were stacked a second time across trials to generate per group templates/reference maps. Combining these images generated a single template per group with a higher confidence interval. (i.e. if a spot is in the template, it is found in all members of that subgroup). This resulted in a given protein spot being more heavily weighted statistically and a lower coefficient of variance per candidate spot, and permitted the combination of the results of similar sub-groups for generating reference/group templates. Combining these data sets increased the power of subsequent statistical analyses and aided in the choice of candidate proteins for identification.

### Heparin effects on selected proteins from cellular compartments

**Figure 3:**
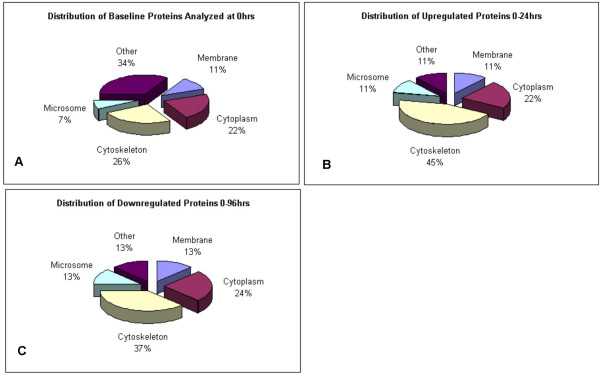
MS identification according to cellular compartment protein expression. **A**. Percentage of the proteins identified by MS in control lungs with respect to cell compartment. **B**. Cell compartment distribution of identified increased proteins in heparin-treated lungs. **C**. Distribution of identified reduced proteins in heparin-treated lungs.

The selected protein peptides analyzed by Mass Spectrometry were classified based on the predicted cellular compartmentalization of the native protein (Fig. 3). It is interesting to note that the percentage of cytoskeleton-related proteins almost doubled among those proteins increased by heparin (Figs. 3A–C). It is understood that some of the larger spots may have masked or otherwise obscured some regulated proteins. However, due to MS limitations of the technique applied in this study (MALDI-TOF/TOF-MS), these proteins spots were not good candidates for quantitative analysis. But it was the purpose of the study to examine those discrete and identifiable proteins whose change in expression exceeded an established threshold (see Methods).

### Overall protein expression changes at 24 hours

A total of 25 candidate proteins were identified as increased at 24 hours post-heparin treatment and an equal number of candidate proteins were selected due to their significant down-regulation pattern or unchanged status, based on comparisons with the control group gels (Fig. 2). Image analysis confirmed these trends on the pixel level. Protein quantity change was considered significant if expression change was greater than 1.7-fold (p < 0.5) (Tables 1 and 2) and CV% were less than 0.1. One of the advantages of separating proteins on 2D gels is the ability to cross-examine MS identifications with protein mobility characteristics. Therefore, the combined results from the image analysis and the MS identification were confirmed by the agreement of mass and iso-electric focusing values calculated via Swiss PDB for the 21 identified proteins satisfying the significance criteria (Tables 1 and 2).

**Table 1:** Increased protein expression profiles

Protein Identified	Accession #	M_r_/D	pl	Magnitutde of expression change					0.96 hr CV%	Protein score
						
				0.24 hr	0.24 hr CV%	24.96	24.96 hr CV%	0.96		
*Parkinson disease prot.7/Drosophila prot. J-1 PARK-7/DJ-1*	gi|16924002	19961.4	6.32	30.05*	0.03	0.34	0.03	10.19*	0.03	108
*Annexin V*	gi|1421099	10028.3	5.3	27.00*	0.12	0.48	0.03	13.02*	0.02	312
*Rat tumor suppressor p30/Hyaluronic acid binding protein 1 homologue HABP-1*	gi|34097946	61034.1	6.2	15.02*	0.05	0.73	0.05	11.00*	0.05	77
*Heat shock protein k71 mutant w ATpase*	gi|1943516	41866.6	6.36	6.44*	0.03	0.5	0.02	3.24*	0.03	92
*Annexin VI*	gi|13994159	75706.5	6.39	6.22*	0.02	0.5	0.02	3.10*	0.01	313
*Septin 2*	gi|16924010	41566.3	6.15	4.19*	0.02	0.24	0.02	1.00	0.02	118
*Capping G protein*	gi|18605629	38744.7	6.47	4.18*	0.02	0.24	0.03	1.00	0.02	375
*Actin related protein 3 Actr-3*	gi|34879484	55252.4	6.55	4.14*	0.02	0.5	0.02	2.07*	0.01	310
*Proteosome 28 subunit(PA28)/Interferon gamma inducible protein (IGIP)*	gi|8394088	28559.1	5.77	3.98*	0.01	0.62	0.03	2.48*	0.02	228
*Moesin*	gi|13540689	6769.8	6.16	3.12*	0.01	0.46	0.01	144	0.01	496
*Rho GDP dissociation inhibitor (Rho-GDI)*	gi|56541074	23392.8	6.12	2.93*	0.02	0.47	0.01	1.38	0.02	77
*Calcyclin/S100A6*	gi|13506725	10028.3	5.3	2.71*	0.03	0.03	0.08	0.09*	0.01	121
*Carboxylesterase 3*	gi|31542380	62107.7	6.1	2.24*	0.05	0.2	0.3	0.45*	0.03	205
*Phosphatidyl Inositol transfer protein (PITPN)*	gi|6679337	31873	5.97	2.03*	0.02	0.44	0.01	0.90	0.02	93
*Heat shock protein 70_90 chaperone*	gi|20302113	62530.4	6.4	2.00*	0.02	0.23	0.02	0.46*	0.02	271
*Proteosome Subunit macropain alpha (PSMA-1)*	gi|38328483	29498.8	6.15	1.99*	0.2	0.51	0.01	0.99	0.02	223

* significant change compared with untreated controls (p < 0.05; or greater than 1.7 fold increase)

**Protein score **– A "score" based on the frequency of a fragment molecular weight found in a protein of a given range of molecular weight. For every database entry scanned, all matching fragments contribute to the final score.

**Protein C.I.% **– Confidence interval that the program assigns to the peptide matches to protein. >95% score was considered statistically significant.

**Table 2:** Reduced protein expression profiles

Protein Identified	Accession #	M_r_/D	pl	Magnitude of expression change					0.96 hr CV%	Protein score	protein C.I.%
							
				0.24 hr	0.24 hr CV%	24.96	24.96 hr CV%	0.96			
*Transgelin 2 homologue (Sm22 alpha)*	gi|34880944	29950.9	8.72	14.00*	0.02	1.00	0.11	14.0*	0.07	114	100.00
*Pyruvate dehydrogenase lipoamide beta*	gi|50925725	38957	6.2	7.50*	0.02	0.75	0.05	5.63*	0.01	119	100.00
*CAPPA1 homologue*	gi|34859736	32889.3	5.43	3.47*	0.05	4.05	0.05	14.4*	0.03	375	100.00
*Lactate dehydrogenase b*	gi|6981146	36589.1	5.7	3.08*	0.02	1.46	0.01	4.51*	0.02	583	100.00
*Dimethylarginine Dimethylaminohydrolase 2 (DDAH-2)*	gi|46237608	29669.4	5.66	2.75*	0.03	0.86	0.04	2.36*	0.07	203	100.00

* significant change compared with untreated controls (p < 0.05; or greater than 1.7 fold increase)

**Protein score **– A "score" based on the frequency of a fragment molecular weight found in a protein of a given range of molecular weight. For every database entry scanned, all matching fragments contribute to the final score.

**Protein C.I.% **– Confidence interval that the program assigns to the peptide matches to protein. >95% score was considered statistically significant.

### Specific groups of proteins changed by heparin at 24 hours

Proteins increased at 24 hours post-heparin treatment fell into four major categories: 1) calcium signaling pathways, 2) cytoskeletal domains, 3) immune responses, and 4) tumor suppression.

**Figure 4:**
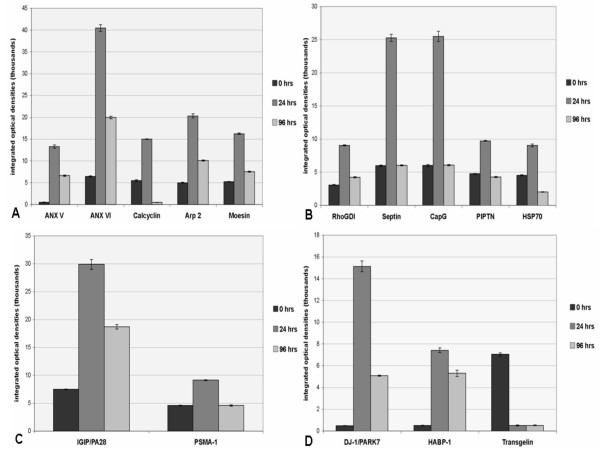
Graphs of protein spot densities represent the time course of heparin's effects on levels of proteins with the greatest change in expression, as detected in 2D gels and identified by MS, due to heparin instillation in rat lung. **A**. Calcium signaling pathway proteins indicated increases in ANXV, ANXVI, calcyclin, ARP3, and moesin (Msn) at 24 hours. While by 96 hours all were reduced from 24 hour levels, only calcyclin was reduced to below 0 hour control levels. **B**. Cytoskeletal domain proteins RhoGDI, septin, CapG, PIPTN, and HSP70 were increased at 24 hours. All decreased to control levels (0 hours) or less by 96 hours. **C**. Two immune response-related proteins, IGIP/PA28 and PSMA-1, were increased at 24 hours, and decreased by 96 hours. **D**. Two proteins relating to tumor suppression, DJ-1/PARK-7 and HABP-1, were increased at 24 hours and reduced by 96 hours, while Transgelin was reduced from control levels at 24 hours and remained low through 96 hours after heparin treatment. Note that Arp3 and Msn are found in the cytoskeletal domain as well as being grouped with the calcium signaling proteins but were graphed only once in A.

A cluster of proteins related to calcium signaling pathways was found to be up-regulated following heparin treatment. Proteomic analysis identified five proteins as up-regulated: annexin V (ANXV) by >27-fold, annexin VI (ANXVI) by > 7 fold, actin related protein 3 (Arp3) by >4-fold, moesin (Msn) by >3 fold, and calcyclin (Cacy/S100A6) by >2.5-fold (Fig. 4A and Table 1).

**Figure 5:**
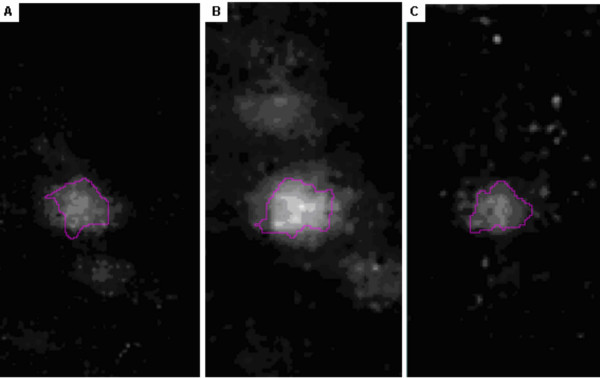
Illustrated is the expression of Rho-GDI protein stained with Sypro-Ruby across all master group-reference maps. **A**. Control, **B**. 24 hrs, and **C**. 96 hrs post-treatment. The expression of Rho-GDI increased 3+ fold from A to B.

Of the cytoskeleton domain proteins, Rho-GDP dissociation inhibitor (Rho-GDI) protein, a key regulator of the Rho-GTPase family was increased by >3-fold; septin and capping G (CapG), two proteins known to be involved in actin filament polymerization, were increased by >5 fold at 24 hours (Fig. 4B and Table 1). In addition, 2-phosphatidyl inositol phosphate transfer protein (PIPTN), a member of the phosphatidyl-inositol-phosphate(PIP)/inositol-phosphatidyl-3 (IP3) family, was increased by >2-fold and Hsp70 doubled (Fig. 4B and Table 1). The change in protein expression as a function of optical density of the electrophoretically resolved spot is further demonstrated by Rho-GDI in an enhanced image (Figs. 5). The increase in spot density from control/saline treatment (Fig. 5A) to heparin treatment at 24 hours (Fig. 5B) and 96 hours (Fig. 5C) is visually evident.

Changes in the expression of two proteins related to immune function were also reflected in the 24 hour post-treatment proteome maps. Interestingly, interferon gamma-inducible proteosome regulator IGIP/PA28, an important modulator of immunoproteosome formation and function, was increased nearly 4-fold (Fig. 4C and Table 1). Proteosome subunit macropain alpha (PSMA-1), known to bind and activate proteosomes critical to immune response in the cytosol and nucleus of cells, was increased nearly 2-fold (Fig. 4C and Table 1).

**Figure 6:**
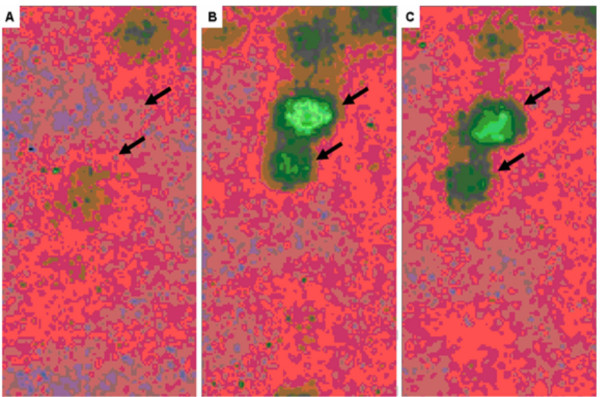
Pseudocolor density image, of in-gel image comparison of expression change for DJ-1/PARK-7 protein across all group maps. **A**. Control, **B**. 24 hrs, **C**. 96 hrs.

Drosophila protein J-1/Parkinson protein 7 (DJ-1/PARK7), a negative regulator of PTEN tumor suppressor and a potential prognostic marker for cancer, was increased by >30-fold at 24 hours and the tumor suppressor-related protein, hyaluronate binding protein-1 (HABP-1), was increased >15 fold (Fig. 4D and Table 1) while transgelin (TAGLN2), was the most significantly down-regulated protein at >14-fold (Fig. 4D and Table 2). The change in DJ-1/PARK7 expression is further demonstrated by in-gel enhancement through computer analysis from control/saline treatment (Fig. 6A) compared to heparin treatment at 24 hours (Fig. 6B) and 96 hours (Fig. 6C).

### Immunostaining for annexin V

**Figure 7:**
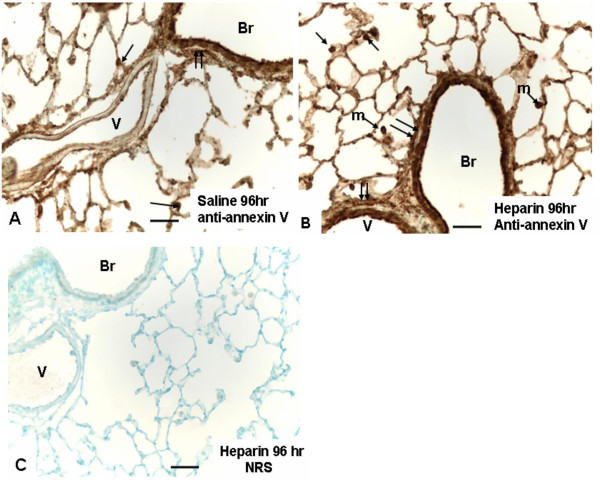
Light micrographs depict representative histologic fields from the various treatment groups indicated **(A-C)**. A. Annexin V immunostaining is seen in bronchiolar (Br) lining epithelium and associated fibroblast layer (double arrows) and alveolar type II epithelial cells (single arrows). Annexin V is also demonstrated in endothelium in pulmonary vessel (V). Magnification ×300. Bar = 30 μm. **B**. Annexin V immunostaining from a heparin-treated lung shows similar features to A, but with generally an increase in overall staining, including macrophages (m), not depicted in panel A. Magnification ×300. Bar = 30 μm. **C**. Histochemical control treated with normal rabbit serum (NRS) in place of rabbit anti-annexin V. Magnification ×200. Bar = 50 μm.

In order to gain some insight into the cellular localization of upregulated proteins, annexin V was chosen as a representative example. Figure 7 shows the immunoreactivity for annexin V in fibroblasts, airway epithelium, macrophages, and alveolar type II cells in the normal rat lung (Fig. 7A). Heparin treatment enhanced immunostaining somewhat (Fig. 7B), with the understanding that immunoreactivity is difficult to truly quantify. Normal serum controls developed no reactive contrast (Fig. 7C).

### Overall protein expression changes at 96 hours

Lungs at 96 hours post-heparin clearly showed a decrease of many proteins compared to the 24 hours post-heparin group. As with the 24 hour analysis, protein identifications were further confirmed by their agreement with the separation patterns and the M_r _calculations performed using the Swiss PDB tool (Tables 1 and 2). There was minimal statistical difference between the treatment sub-groups in expression of the selected proteins, with most being reduced from the 24 hour period. However, most remained elevated above control levels. Those remaining significantly increased at the 96 hour post-heparin time point were ANXV, ANXVI, Arp3 (Fig. 4A and Table 1), IGIP/p28 (Fig. 4C and Table 1), and HABP-1, and DJ-1/PARK7 (Fig. 4D and Table 1).

## Discussion

This report describes the 2D gel electrophoresis and MS analysis of selected proteins in normal and heparin-treated rat lung and identifies proteins that change in quantity 24 and 96 hours after a single in-vivo aerosol exposure to heparin. The twenty most dynamically expressed proteins were selected at the time points tested in the study and further analyzed by MS. They represent potential biomarkers or indicators of cellular/tissue responses to heparin and sort into specific categories that could be useful in a systems biology approach to create a mechanistic model of heparin's actions on whole rat lung.

### Calcium signaling pathways

Based on lung proteome analysis, ANXV, which is a calcium- and phospholipid-binding protein, had the second greatest increase (>27-fold change at 24 hours) and the greatest increase overall by 96 hours (>13-fold). Annexins have previously been shown to bind to GAG's in the presence of calcium. Perhaps relatedly, calcyclin (S100A6), another calcium-binding protein, was increased nearly 3-fold at 24 hours post-heparin. Calcyclin is known to bind to ANXV in the presence of calcium [18]. The calcyclin+Ca+ANXV complex has been shown to be involved in actin polymerization via the Arp2/3 complex [18-20]; Arp3 was one of the identified proteins increased in this proteomic analysis. Actin polymerization could subsequently enhance the dynamic relocation of cytoskeleton-linking attachment proteins [19] such as moesin, also observed to be increased in this study. These cumulative observations support the notion that heparin treatment of whole lung as in the present study and as has been previously reported in cell cultures [19,21] results in calcium influx into lung cell cytosolic compartments and triggers significant increases in expression or relocation of selected groups of proteins related to cytoskeletal reorganization and immune function.

### Cytoskeletal domains

It has been demonstrated that inhaled heparin can prevent exercise-induced bronchoconstriction in asthma patients [15,17], and there is evidence suggesting heparin's potential in promoting wound healing [22-24] and in preventing lung fibrosis [13,25,26], although the underlying mechanisms involved remain unclear. In this study it was found that a number of cytoskeleton-related proteins were increased at 24 hours post-heparin treatment. Based on bioinformatics and biological data-mining models, these results support the notion that heparin treatment appears to increase selected contractile and structural elements in the lung. Specifically, Rho-GDI protein was found to be increased >3-fold at 24 hours post-heparin. Rho-GDI is known to bind the key activator, Ras, which actuates important cellular events including cellular growth, movement, apoptosis, and immune regulation. The binding is known to be inhibitory of Rac, a small GTPase involved in cytoskeleton contractility [27]. Both events might be expected to reduce/prevent contractile events, such as bronchoconstriction. This same inhibition may promote cellular adhesion events and cytoskeletal stability. Perhaps relatedly, other contractile/cytoskeletal proteins were also found to be increased by heparin. Moesin and PIPTN were up-regulated >3-fold and >2-fold, respectively, by 24 hours. Moesin and PIPTN enable the cytosolic RhoA/Rho-GDI complex to partially unfold and reveal hydrophobic regions which are able to attach to cell membrane surfaces, and in doing so, release Rho-GTP proteins – active in cytoskeletal reorganization [28-33]. Moesin, a significant attachment protein, binds to the cell surface HSPG, syndecan-2 [34]. In addition to being implicated in cell migration and actin cytoskeletal reorganization, PIPTN – also identified as increased >2-fold by heparin – has been shown to independently activate NADPH protein [35]. Receptor stimulation transmitted by Rho and PIPTN activates proteins at the leading edge of migrating cells and recruits other actin-related proteins, such as Arp3 (also increased >4-fold by heparin) in complex with Arp2, and actin monomers, to induce actin filament branching and membrane protrusion. This can be accomplished through activation of scaffold proteins called WASp's, which are capable of activating the Arp2/3 complex and promoting actin cytoskeleton polymerization [36]. Polymerization has been shown to accelerate when WASp is in the presence of septin, another protein increased by heparin treatment in the present study (Fig. 3B). It has also been suggested that there are direct relationships among septin, the actin cytoskeleton, and the Rho family of proteins [37]. Another protein increased by heparin was heat shock protein 70, HSP70 (~2-fold), which mediates cytoprotective effects through its function as a molecular chaperone and through the phosphorylation-dependent stabilization of actin filaments [38].

It's worth noting that the cell type(s) and their numbers with increased expression of cytoskeletal proteins is not known from these studies. Smooth muscle cells and the transition of interstitial fibroblasts to myofibroblasts might be logical sources of the observed increases. Early work suggested that heparin induces smooth muscle actin expression and differentiation of myofibroblasts [39]. More recent studies suggest that heparin in combination with fibroblast growth factors prevent myofibroblast differentiation [40,41] – perhaps more likely a scenario to be expected in an in vivo environment. Expansion of smooth muscle is an even less likely source, since heparin is well recognized for its antiproliferative effects on these cells. Future studies will hopefully clarify this important point and area of investigation.

### Immune response

There is increasing evidence for the anti-inflammatory effects of heparin [5,6,12,42-45]. The current study supports this body of evidence by reporting heparin's effects on the expression of two proteins important in the immune response. IGIP/PA28 plays key roles in immune response mechanisms [46,47] and has been shown to act in intracellular immune functions via cytoskeletal membrane trafficking and processing, possibly through its binding to Hook3 protein, an actin-microtubule-binding protein [48]. In concert with the combination of several regulatory events, IGIP/PA28 aids in production of MHC class 1 antigen in interferon gamma-stimulated cells [49,50]. Interestingly, heparin and/or IGIP/PA28 also stimulate STAT-1 and STAT-3 pathways, which are anti-inflammatory in a number of cell models [51,52]. The second protein, PSMA-1, along with IGIP/PA28, activates proteosomes which produce antigenic peptides required for the stimulation of immune responses [50].

### Tumor suppression

DJ-1/PARK7, a negative regulator of the tumor suppressor PTEN and promoter of proliferative pathways and cell survival [53,54], was increased (>30-fold) at 24 hours. It's not clear how this reconciles with heparin's established anti-proliferative effects on cells in general, but may reflect a more specific response that remains to be illucidated. Heparin's known anti-tumor effects may be reflected in our observation that, at 24 hours post-heparin treatment, the tumor suppressor protein HABP-1 is increased >15-fold. In addition, transgelin, an actin binding cytoskeletal protein known also to have a role in calcium interactions of smooth muscle cells, was the most significantly reduced protein at 14-fold. Transgelin is associated with cellular changes in idiopathic pulmonary fibrosis (IPF) and was previously proposed to be a candidate tumor antigen [55-58]. The mechanisms for these changes present intriguing targets for future studies.

### Overall protein expression changes at 96 hours

It is important to note that nearly half of the proteins detected which related to cytoskeletal reorganization return to nearly baseline/control levels of expression by 96 hours. There was a general decrease in protein expression by 96 hours post-treatment compared to the 24 hours time-point. It is likely that the effects of heparin on most cells decrease with time, since the animals were not retreated. This is consistent with other in vivo and in vitro studies from our lab. Other proteins, like ANXV, ANXVI, IGIP/PA28, and HABP-1, were increased at 24 hours and remained highly elevated at 96 hours – and indication that heparin has more extended effects on selected proteins.

## Conclusion

In the present study, alterations of the rat lung proteome in response to heparin are detailed for the first time. The majority of proteins that were up-regulated were related to calcium signaling and cytoskeletal organization, as evidenced by the strong increases in ANXV and ANXVI, Msn, RhoGDI, PIPTN, calcyclin, and Arp3. These may serve as potential biomarkers or indicators of baseline response to changes in the lung macro- and/or microenvironments, aiding in further understanding of the lung proteome, in addition to understanding specific organ responses to challenge or stimulation. This, and the indication that other major groups of proteins related to immune function and control of cell growth were also responsive to heparin treatment, suggests that heparin's known suppressive effects upon lung fibrosis and even some cancers may result from its effects upon cell shape and motility, moderation of cell growth, and stimulation of the immune response. This approach may prove useful to future systems biology studies addressing changes to tissue architecture of lung, such as asthma, chronic obstructive lung disease, and cystic fibrosis. Further, these results also raise the possibility that shed surface ectodomains of proteoglycans, as modeled in this study by heparin, may have a broader influence on biological responses in the lung than previously appreciated. Future studies will help clarify this potentially important issue.

## Competing interests

The author(s) declare they have no competing interests.

## Authors' contributions

AG and PS were involved in the concept and design of all experiments, and AG, PS, MR, JP and DN were involved in analysis and interpretation of data. MR, JP, and JK were involved in data acquisition and further analysis. AG and JK were directly responsible for the animal experimentation, AG performed all protein separations, MR and JP performed all mass spectroscopy, and AG analyzed and formatted all data sets. AG, PS, and DN coordinated and prepared the manuscript, which was further edited by MR and JP. All the authors read and approved of the manuscript.
